# Supplementary material for: Adaptive Mechanisms of Renal Bile Acid Transporters in a Rat Model of Carbon Tetrachloride-Induced Liver Cirrhosis
Source: J Clin Med. 2022 Jan 27;11(3):636. doi: 10.3390/jcm11030636 (PMC8836491; doi:10.3390/jcm11030636)
Supplement: Supplementary file 1 [file jcm-11-00636-s001.zip › jcm-1542443-supplementary.pdf]

Table S1. Technical characteristics of the antibodies used for immunohistochemistry.

Antibody	Clone	Manufacturer	Dilution
NTCP	*Rabbit Polyclonal*	AbCam [330 Cambridge Science Park Cambridge CB4 0FL UK]	1:250
BSEP	*Rabbit Polyclonal*	AbCam [330 Cambridge Science Park Cambridge CB4 0FL UK]	1:200
P-Gly	*Rabbit Polyclonal*	AbCam [330 Cambridge Science Park Cambridge CB4 0FL UK]	1:100
ASBT	*Rabbit Polyclonal*	Bioss [Schloss-Rahe-Str. 15 52072 Aachen Germany]	1:100
MRP-4	*Rabbit Polyclonal*	AbCam [330 Cambridge Science Park Cambridge CB4 0FL UK]	1:200
Osteopontin	*Rabbit Polyclonal*	Antibodies online [Dunwoody Park, Suite 145 - Atlanta, GA 30338 - US]	1:100
Caspase-3	*Rabbit Polyclonal*	AbCam [330 Cambridge Science Park Cambridge CB4 0FL UK]	1:50
TLR-4	*Rabbit Polyclonal*	Lifespan Bioscience, UK	1:100

NTCP, solute carrier family 10 member; BSEP, bile salt export pump; P-Gly, P-Glycoprotein; ASBT, solute carrier family 10 member 2; MRP4, multidrug resistance protein 4; TLR-4, Toll Like Receptor 4.

Table S2. Liver histology score according to the scoring systems developed by Ishak et al. [1].

Modified HAI grading: Necroinflammatory scores							
**Periportal or periseptal interface hepatitis (piecemeal necrosis)**	**Score**	**Confluent necrosis**	**Score**	**Focal (spotty) lytic necrosis, apoptosis and focal inflammation**	**Score**	**Portal inflammation**	**Score**
Absent	0	Absent	0	Absent	0	None	0
Mild	1	Focal confluent necrosis	1	One focus per 10x objective	1	Mild,	1
Mild/moderate	2	Zone 3 necrosis in some areas	2	Two to four foci per 10x objective	2	Moderate,	2
Moderate	3	Zone 3 necrosis in most areas	3	Five to ten foci per 10x objective	3	Moderate/marked	3
Severe	4	Zone 3 necrosis / occasional portal-central bridging	4	More than ten foci per 10x objective	4	Marked	4
		Zone 3 necrosis + multiple bridging	5				
		Panacinar or multiacinar necrosis	6				
*Does not include diffuse sinusoidal infiltration by inflammatory cells							

Table S3. Staging, architectural changes and fibrosis according to the scoring systems developed by Ishak et al. [1] and to METAVIR (Meta-analysis of Histological Data in Viral Hepatitis) [2].

**Fibrosis score following two scoring systems**				
**Ishak's scheme [1]**		**METAVIR system [2]**		**Comparison**
**Change**	**Score**	**Description**	**Score**	
No fibrosis	**0**	No fibrosis	**0**	**0**
Fibrous expansion of some portal areas, with or without short fibrous septa	**1**	Stellate enlargement of portal tract but without septa formation	**1**	**1 vs 1**
Fibrous expansion of most portal areas, with or without short fibrous septa	**2**	Stellate enlargement of portal tract but without septa formation	**1**	**2 vs 1**
Fibrous expansion of most portal areas, with occasional portal to portal (P-P) bridging	**3**	Enlargement of portal tract with rare septa formation	**2**	**3 vs 2**
Fibrous expansion of portal areas with marked bridging [portal to portal (P-P) as well as portal to central (P-C)]	**4**	Numerous septa formation	**3**	**4 vs 3**
Market bridging (P-P and/or P-C) with occasional modules (incomplete cirrhosis)	**5**	Numerous septa formation	**3**	**5 vs 3**
Cirrhosis, probable or definite	**6**	Cirrhosis	**4**	**6 vs 4**

Table S4. Serum creatinine (mg/dL) of rats during cirrhosis induction at week 1 (T1), week 2 (T2), week 4 (T4), week 8 (T8) and week 12 (T12). Creatinine of control rats was steadily normal (< 0.17 mg/dL).

ID	T1	T2	T4	T8	T12
1	< 0.17	< 0.17	0.18	0.19	0.44
2	< 0.17	< 0.17	0.24	< 0.17	0.35
3	0.18	< 0.17	0.26	< 0.17	0.19
4	< 0.17	< 0.17	0.26	< 0.17	0.47
5	< 0.17	< 0.17	0.28	< 0.17	0.30
6	< 0.17	< 0.17	< 0.17	< 0.17	0.29
7	< 0.17	< 0.17	0.23	0.26	0.22
8	< 0.17	< 0.17	0.21	< 0.17	0.22
9	< 0.17	< 0.17	< 0.17	< 0.17	0.38
10	< 0.17	< 0.17	< 0.17	< 0.17	0.53
11	< 0.17	< 0.17	n.d.	< 0.17	0.36
12	< 0.17	< 0.17	< 0.17	< 0.17	0.24
13	< 0.17	< 0.17	< 0.17	< 0.17	0.57
14	< 0.17	< 0.17	< 0.17	< 0.17	0.56
15	< 0.17	< 0.17	< 0.17	0.25	0.40
16	< 0.17	< 0.17	< 0.17	0.25	0.48
17	< 0.17	0.22	< 0.17	0.18	0.35
18	< 0.17	< 0.17	< 0.17	0.17	0.44
19	< 0.17	< 0.17	< 0.17	0.20	0.23
20	< 0.17	< 0.17	< 0.17	0.19	0.42
21	< 0.17	0.19	< 0.17	0.26	0.23

Table S5. Serum total bilirubin (mg/dL) of rats during cirrhosis induction at week 1 (T1), week 2 (T2), week 4 (T4), week 8 (T8) and week 12 (T12). Total bilirubin of control rats was steadily normal (< 0.15 mg/dL).

ID	T1	T2	T4	T8	T12
1	< 0.15	< 0.15	< 0.15	< 0.15	2.42
2	< 0.15	< 0.15	< 0.15	< 0.15	3.49
3	< 0.15	< 0.15	< 0.15	0.16	2.74
4	< 0.15	< 0.15	< 0.15	< 0.15	< 0.15
5	< 0.15	< 0.15	< 0.15	< 0.15	3.76
6	< 0.15	< 0.15	< 0.15	< 0.15	1.88
7	< 0.15	< 0.15	< 0.15	< 0.15	< 0.15
8	< 0.15	< 0.15	< 0.15	< 0.15	0.59
9	< 0.15	< 0.15	< 0.15	< 0.15	< 0.15
10	< 0.15	< 0.15	< 0.15	< 0.15	< 0.15
11	< 0.15	< 0.15	n.d.	< 0.15	< 0.15
12	< 0.15	< 0.15	< 0.15	< 0.15	0.63
13	< 0.15	< 0.15	< 0.15	< 0.15	< 0.15
14	< 0.15	< 0.15	< 0.15	< 0.15	1.26
15	< 0.15	< 0.15	< 0.15	< 0.15	2.11
16	< 0.15	< 0.15	< 0.15	< 0.15	1.6
17	< 0.15	< 0.15	< 0.15	< 0.15	< 0.15
18	< 0.15	< 0.15	< 0.15	< 0.15	0.4
19	< 0.15	< 0.15	< 0.15	< 0.15	2.23
20	< 0.15	< 0.15	< 0.15	< 0.15	< 0.15
21	< 0.15	< 0.15	< 0.15	< 0.15	< 0.15

References

1. Ishak K, Baptista A, Bianchi L, Callea F, De Groote J, Gudat F, Denk H, Desmet V, Korb G, MacSween RN, et al. Histological grading and staging of chronic hepatitis. J Hepatol. 1995 Jun;22(6):696-9. doi: 10.1016/0168-8278(95)80226-6.
2. Bedossa P, Poynard T. An algorithm for the grading of activity in chronic hepatitis C. The METAVIR Cooperative Study Group. Hepatology. 1996 Aug;24(2):289-93. doi: 10.1002/hep.510240201.
